# Anti-Inflammatory and Protective Effects of Water Extract and Bioferment from *Sambucus nigra* Fruit in LPS-Induced Human Skin Fibroblasts

**DOI:** 10.3390/ijms241210286

**Published:** 2023-06-17

**Authors:** Magdalena Wójciak, Aleksandra Ziemlewska, Martyna Zagórska-Dziok, Zofia Nizioł-Łukaszewska, Dariusz Szczepanek, Tomasz Oniszczuk, Ireneusz Sowa

**Affiliations:** 1 Department of Analytical Chemistry, Medical University of Lublin, Chodzki 4a, 20-093 Lublin, Poland; magdalena.wojciak@umlub.pl; 2 Department of Technology of Cosmetic and Pharmaceutical Products, Medical College, University of Information Technology and Management in Rzeszow, Kielnarowa 386a, 36-020 Tyczyn, Poland; aziemlewska@wsiz.edu.pl (A.Z.); mzagorska@wsiz.edu.pl (M.Z.-D.); zniziol@wsiz.edu.pl (Z.N.-Ł.); 3 Chair and Department of Neurosurgery and Paediatric Neurosurgery, Medical University of Lublin, 20-090 Lublin, Poland; dariusz.szczepanek@umlub.pl; 4 Department of Thermal Technology and Food Process Engineering, University of Life Sciences in Lublin, Głęboka 31, 20-612 Lublin, Poland

**Keywords:** elderberry, polyphenols, metalloproteinases, kombucha, interleukins, skin cells, antioxidants

## Abstract

In this study, an attempt was made to evaluate the antioxidant, anti-inflammatory and protective effects of the *Sambucus nigra* fruit extract and its ferment obtained by fermentation with kombucha tea fungus. For this purpose, fermented and non-fermented extracts were compared in terms of their chemical composition by the HPLC/ESI-MS chromatographic method. The antioxidant activity of the tested samples was assessed using DPPH and ABTS assays. Cytotoxicity was also determined using Alamar Blue and Neutral Red tests to assess the viability and metabolism of fibroblast and keratinocyte skin cells. Potential anti-aging properties were determined by their ability to inhibit the activity of the metalloproteinases collagenase and elastase. Tests showed that the extract and the ferment have antioxidant properties and stimulate the proliferation of both cell types. The study also assessed the anti-inflammatory activity of the extract and ferment by monitoring levels of the pro-inflammatory interleukins IL-6, IL-1ß, tumor necrosis factor (TNF-α) and anti-inflammatory IL-10 in lipopolysaccharide (LPS)-treated fibroblast cells. The results indicate that both the *S. nigra* extract and its kombucha ferment can be effective in preventing free-radical-induced cell damage and have positive effects on skin cell health.

## 1. Introduction

In recent years, interest in medicinal plants has been constantly increasing and many scientific studies are being conducted to reveal their health-promoting effects. They are valuable sources of biologically active compounds, among which polyphenols deserve special attention due to their multidimensional activities and strong antioxidant properties [1]. They serve as additives to foods and cosmetics to enhance their beneficial effects.

Berries of *Sambucus nigra* (elderberry) belonging to the Adoxaceae family are commonly considered as a rich source of polyphenolic compounds and are widely utilized in beverages as well as in the production of jams and jellies and functional food [2,3]. In addition, elderberry has a long history of use in folk medicine and has been applied as a herbal remedy for colds, flu, and respiratory tract dysfunction [4,5].

**Scheme 1:**
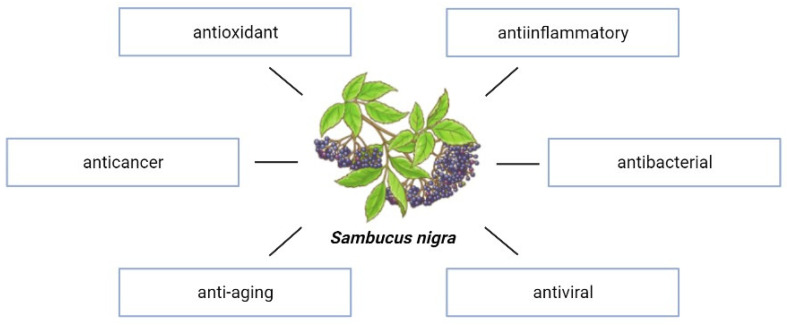
Representation of the main cosmetic and therapeutic effects of *Sambucus nigra*.

Many scientific studies confirm the potent benefits of elderberry in supporting the immune system, reducing inflammation, and supporting cardiovascular health. Furthermore, it has been evidenced that it exhibits antioxidant, antibacterial, and antiviral properties [6,7,8,9]. Some of the aforementioned features make it a desirable ingredient of cosmetics formulations (Scheme 1). As indicated by the literature data, biologically active polyphenolic compounds, such as gallic acid, chlorogenic acid, catechin, quercetin, cyanidin 3-sambubioside and cyanidin 3-glucoside, are responsible for the health-promoting properties of the tested extract [10,11,12]. Gallic acid has been shown to accelerate wound healing by protecting skin cells from oxidative stress and activating FAK, JNK and Erk in human keratinocytes. More importantly, gallic acid significantly improves wound healing under hyperglycemic conditions by promoting cell migration [10,11,12,13]. Catechins, in turn, are characterized by antioxidant, anticancer, and antiviral properties [14]. Extensive research into the UV-protective capacity of catechins has shown that they are able to enhance photostability and skin protection against UV rays [15]. Chlorogenic acid is a powerful antioxidant and has been shown to have anti-aging and photoprotective properties [16]. Quercetin also shows many promising health effects for the skin, such as anti-inflammatory, anti-aging, antimicrobial, antiviral, anticancer, anti-osteoporosis, antifungal, anti-psoriasis, wound healing, anti-itching, skin whitening and photoprotection [17].

Anti-inflammatory and antioxidant actions have great significance in skin protection and in delaying skin aging. Senescent cells lose the ability to proliferate but remain metabolically active, and they affect the skin’s microenvironment through the secretion of proteases and proinflammatory factors. Thus, skin aging is accompanied by chronic inflammation, resulting in increased levels of inflammatory cytokines such as IL-6, IL-8, IL-18 and TNF-α. This condition is referred to as “inflammaging.” Furthermore, the extracellular matrix (ECM) is remodeled, and the disrupted homeostasis of matrix metalloproteinases (MMPs) promotes age-associated changes in the skin’s architecture and function [18]. In addition, the concentration of reactive oxygen species (ROS) increases in aging skin cells, leading to DNA damage, fragmentation and disorganization of collagen fibers, and further progressive structural and functional skin alterations [19]. Therefore, effective exogenous anti-inflammatory and antioxidant substances, including plant extracts, can protect cells and delay unfavorable processes in the skin. 

In our study, the anti-inflammatory and antioxidant activity of elderberry extract was evaluated using human skin fibroblasts and keratinocytes to assess its potential for cosmetic purposes. Its cytotoxicity and effect on enzymes responsible for the degradation of collagen and elastin fibers were also studied.

Furthermore, the impact of fermentation on the properties and chemical composition of elderberry extract was evaluated. This process increases the biological potential of plant material by converting high-molecular compounds into low-molecular ones what enhance their effectiveness and bioavailability [20]. Recently, fermented cosmetics have been gaining increasing importance in skin protection and maintaining skin health [21].

## 2. Results

### 2.1. Phytochemical Investigation and Quantitative Analysis of Main Components

**Figure 1:**
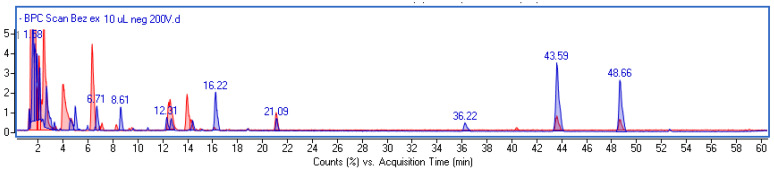
Overlapped base-peak chromatograms (BPC) of *S. nigra* extract (blue line) and fermented extract (red line) obtained in negative ionization mode.

The phenolic composition of the water extract from *S. nigra* fruit was determined using UHPLC-MS. The components were analyzed in negative and positive ionization modes and identified based on mass data (M-H), fragmentation patterns, and UV–Vis spectra (200–600 nm). Representative overlapped chromatograms for the extract and the fermented extract are shown in Figure 1.

Chromatographic analysis revealed qualitative and quantitative differences between the elderberry extract (EB) and the fermented elderberry extract (FEB). The polyphenolic composition of FEB was more diverse and, compared with EB, some additional compounds were observed, including gluconic acid, gallic acid, catechin, neochlorogenic acid, rutin, dihydroxybenzoic and hydroxybenzoic acid glucoside. In both extracts, protocatechuic acid (PA) was the predominant phenolic acid; however, its amount was approximately 5.5-fold higher in the FEB. Moreover, three peaks with characteristic UV–Vis spectrum typical of anthocyanins have been recorded in the EB, with the common ion fragment corresponding to aglycone cyanidin (*m*/*z*-H = 285). Based on the literature [6], they have been identified as cyanidin-sambubioside-5-glucoside, cyanidin 3-sambubioside and cyanidin 3-glucoside. The mentioned components were also ionizable in the positive mode with pseudomolecular ions of *m*/*z* + H 743.20301, 581.15016, and 449.10797, respectively. However, cyanidin 3-sambubioside-5-glucoside was not found in the fermented extract and the amount of cyanidin 3-sambubioside was lower than in EB. Furthermore, a few unidentified peaks were visible on the EB’s BPC chromatogram that had a characteristic MS spectrum (Figure S1) and no UV–Vis absorption. These peaks were significantly lower in FEB. Mass data and results of the quantitative analysis of polyphenols are summarized in Table 1.

### 2.2. Antioxidant Assay

In order to assess the antioxidant properties of the tested samples, two different tests based on different mechanisms of action were used—DPPH and ABTS assays. The DPPH assay is based on the hydrogen-donating capacity to scavenge the DPPH radical. During the reaction, DPPH accepts the electron donated by the antioxidant and loses its purple color, turning yellow in the presence of oxidants. In the ABTS assay, the generation of the cation radical ABTS+ involves the direct generation of the blue-green chromophore ABTS in the reaction between ABTS and potassium persulfate. The radical formed during the reaction is blue-green in color. By reducing the cationic radical, antioxidants cause the color of the solution to fade, with the decrease in color intensity depending on the amount of antioxidants in the solution. The measure of antioxidant activity is the value of the IC_50_ parameter, which determines the concentration of antioxidant that causes a 50% decrease in the initial radical concentration. The IC_50_ results for the DPPH and ABTS assays are shown in Table 2. These values indicate that the samples with the lowest IC_50_ value have the best antioxidant properties. Based on the DPPH and ABTS methods, the *Sambucus nigra* extract has stronger antioxidant properties than its kombucha ferment. However, the differences between the extract and the ferment are not large.

### 2.3. Cytotoxicity Assessment

**Figure 2:**
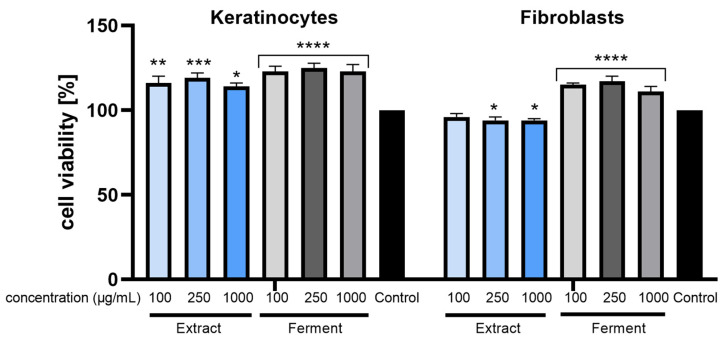
Effect of increasing concentrations of *S. nigra* extract and ferment (100, 250 and 1000 μg/mL) on resazurin reduction in cultured keratinocytes and fibroblasts after 24 h of exposure. Data are the mean ± SD of three independent experiments, each consisting of three replicates per treatment group **** *p* < 0.0001, *** *p* < 0.001, ** *p* < 0.01, and * *p* < 0.05 compared with the control.

**Figure 3:**
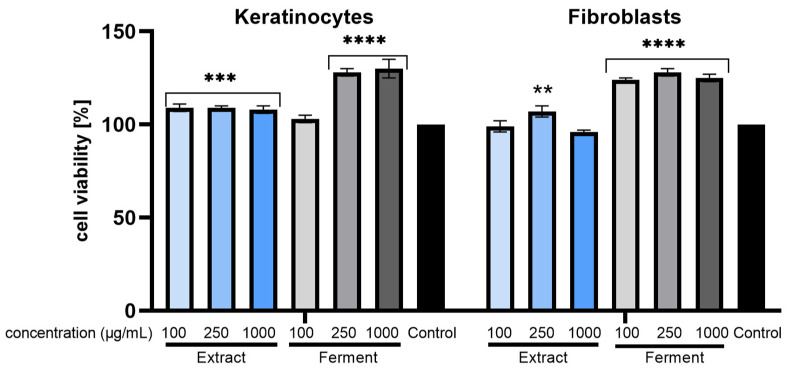
Effect of increasing concentrations of *S. nigra* extract and ferment (100, 250 and 1000 μg/mL) on neutral red uptake in cultured keratinocytes and fibroblasts after 24 h of exposure. Data are the mean ± SD of three independent experiments, each consisting of three replicates per treatment group **** *p* < 0.0001, *** *p* < 0.001, ** *p* < 0.01 compared to the control.

Cytotoxicity studies of the tested extract and ferment were carried out using tests enabling the quantitative estimation of the number of living cells in the culture. The first, Alamar Blue (AB), evaluates the reducing capacity of living cells to convert resazurin to the fluorescent resorufin, while the second, Neutral Red (NR), is based on the ability of living cells to bind the neutral red dye in lysosomes. Two cell lines located in two different layers of the skin were used to assess the cytotoxicity of elderberry extract and the ferment. Keratinocytes (HaCaT cells) represent the major cell type of the epidermis, while fibroblasts (BJ cells) are connective tissue cells found in the dermis. Inflammatory signals activate the proliferation and maturation of these two types of cells, which are essential for wound healing and which play an extremely important role in the repair and regeneration of the skin [22]. Moreover, there is an active interaction between keratinocytes and fibroblasts in maintaining skin homeostasis, and any disruption of this homeostasis can lead to various undesirable skin diseases [23]. The conducted analyses showed no cytotoxic effect of the *S. nigra* extracts and ferments on both keratinocytes and fibroblasts. In the case of the AB test, it was shown that the EB is able to stimulate the activity of keratinocytes, while in the case of fibroblasts, a slight but statistically significant inhibition of cell viability was observed after exposure to two higher concentrations of EB (250 and 1000 μg/mL). More promising results were obtained with the FEB, which increased the viability of both types of cells (Figure 2). In the NR assay, analogous results were obtained, except that the increase in viability of both types of cells after using the EB was negligible, while in the case of the FEB, a significant increase in the viability of both HaCaT and BJ cells was observed (Figure 3).

### 2.4. Assessment of Matrix Metallopeptidase Inhibition

**Figure 4:**
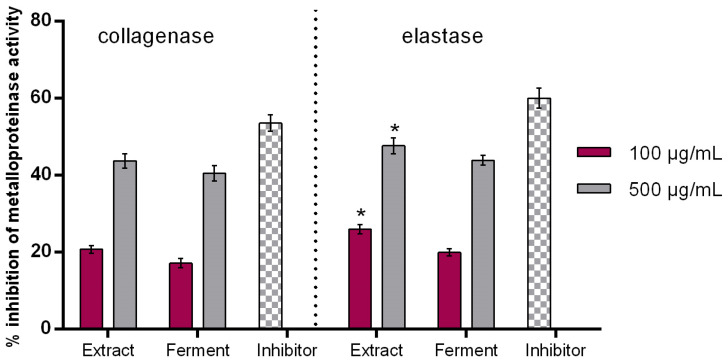
Collagenase and elastase inhibitory activity of *S. nigra* extract and ferment at concentrations of 100 and 500 μg/mL. Data are the mean of three independent experiments, each consisting of two replicates per treatment group. * *p* < 0.05, comparing the extract to the ferment.

To enable the use of elderberry extract and the kombucha ferment in anti-aging preparations, it is important to assess their ability to inhibit the activity of enzymes closely involved in skin aging. The main enzymes whose increased activity leads to the degradation of collagen and elastin fibres that accelerates skin aging are collagenase and elastase [24]. In this study, the inhibition of the activity of the aforementioned enzymes was measured for elderberry extract and the ferment at the two concentrations of 100 and 500 µg/mL, and the results are shown in Figure 4. It was observed that the ability to inhibit both collagenase and elastase was concentration-dependent for the samples tested, with inhibition percentages increasing with the concentration tested. When comparing the percentage of inhibition of collagenase and elastase activity for the extract and the ferment, no statistically significant differences (for collagenase) or small statistically significant differences (for elastase) were observed. The values are comparable for the extract and the ferment and are approximately 20 and 40% (for concentrations of 100 and 500 ug/mL, respectively). The percentages of metalloproteinase inhibition activity were compared with those for commonly used inhibitors. These results suggest that both the plant extract and its kombucha ferment have anti-elastase and anti-collagenase properties.

### 2.5. Protecive Effect of Extracts on LPS-Induced Cytotoxicity 

**Figure 5:**
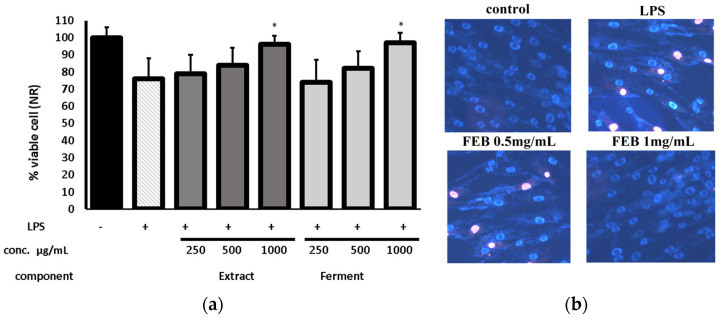
(**a**) Effect of pretreatment with different concentrations of *S. nigra* fruit extract (EB) and fermented extract (FEB) prior to LPS (10 μg/mL) exposure on cell viability as measured by the Neutral Red assay. The data represent means ± SD (*n* = 3). * *p* < 0.05 compared with LPS-treated cells. (**b**) Photographs show the staining of the cells with propidium iodide and Hoechst 33342. Magnification: 200×.

The tests aimed to assess the protective effect of the extract and the ferment against LPS-induced cytotoxicity. The results are shown in Figure 5 and Figure S2.

As can be seen, LPS stimulation reduced the percentage of viable cells to approximately 75% and 70% in the NR and MTT assays, respectively, compared with the control. However, the addition of the extract or fermented extract at a concentration of 1 mg/mL 2 hours before LPS treatment protected the cells from the negative effects of LPS. The differences between the EB and FEB were statistically significant. Figure 5b demonstrates the presence of apoptotic cells in LPS-treated fibroblasts, as shown by the pink fluorescence of the nuclei, which indicates the penetration of this dye into the cells and intercalations between the bases present in the DNA sequence as a result of the death of the tested cells. The cytotoxic effect of LPS was inhibited by pretreatment of the cells with the *S. nigra* ferment at a concentration of 1 mg/mL, as indicated by the blue staining of the chromatin of these cells. The use of a lower concentration of ferment (0.5 mg/mL) was insufficient to eliminate the cytotoxic effect of LPS, as evidenced by the intercalation of propidium iodide into the cells.

### 2.6. Anti-Inflammatory Assay

**Figure 6:**
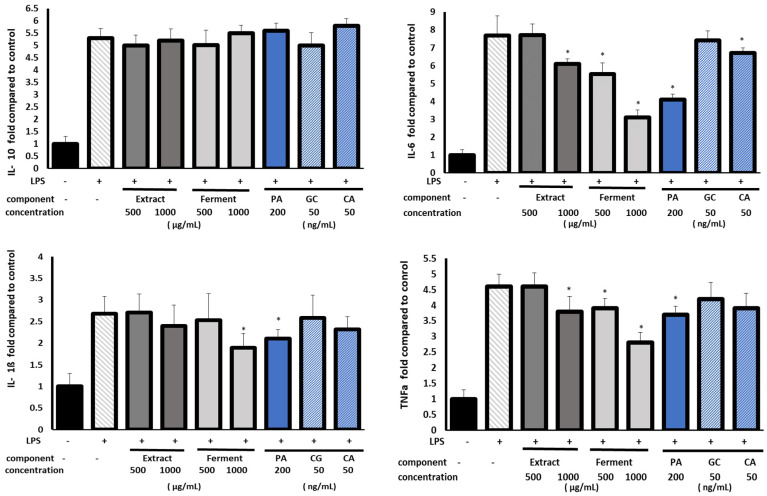
Effect of pretreatment with different concentrations of *S. nigra* fruit extract (EB), fermented extract (FEB), or standard compound solution prior to LPS exposure on interleukin levels, calculated as a percentage compared with the untreated control. The data represent means ± SD (*n* = 3) and was analyzed by one-way ANOVA followed by Dunnett’s multiple comparison post hoc test. * indicates a statistically significant difference (*p* < 0.05) compared with the LPS-treated cells. PA—protocatechuic acid; CG—cyanidin 3-O-glucoside; CA—chlorogenic acid.

To assess the anti-inflammatory activity of the extract, levels of the proinflammatory interleukins IL-6, IL-1β, tumor necrosis factor (TNF-α), and anti-inflammatory IL-10 were monitored in LPS-treated fibroblast cells. Protocatechuic acid, chlorogenic acid, and cyanidin 3-glucoside were also included in the investigation due to the existing literature data on their anti-inflammatory properties. The concentrations of these compounds were chosen based on their calculated content in the fermentation extract broth (FEB) at 1 mg/mL. The results are presented in Figure 6. LPS was a potent inducer of the cytokines, leading to a significant increase in their levels (approximately 2.7 to 7.6-fold). As shown in the results, the tested extracts had no impact on IL-10 levels, while only FEB at 1 mg/mL reduced the level of IL-1β. On the other hand, both the EB and the FEB demonstrated a concentration-dependent reduction in the production of IL-6 and TNF-α. However, the FEB exhibited greater effectiveness. Chlorogenic acid and cyanidin 3-O-glucoside, at concentrations similar to those found in the FEB, did not demonstrate any anti-inflammatory effects. However, they were effective in lowering cytokine levels at higher concentrations. In contrast, protocatechuic acid suppressed the levels of the proinflammatory cytokines IL-6, IL-1β, and TNF-α. This suggests that PA may be responsible for the anti-inflammatory action of the FEB. However, PA at the concentration found in the fermented extract exhibited lower activity compared to the FEB itself, indicating a synergistic effect of FEB components.

## 3. Discussion

Interest in natural ingredients as additives to cosmetic formulations has recently increased, as it is believed that they are valuable agents that enhance health-promoting properties [25]. In our study, the impact on cell viability and cell metabolism and skin metalloproteinase as well as the antioxidant and anti-inflammatory effects of elderberry extract (EB) were investigated. Furthermore, the influence of the fermentation process on the chemical composition and biological activity of the extract was studied. 

In our work, the extract was prepared using water. Although this solvent is not very effective for isolating various phenolic compounds, the water extract is safe for cells and microorganisms and can be subjected to fermentation. Kombucha, which is the mixture of a symbiotic culture of bacteria from the *Acetobacter* and Gluconobacter genera and yeast belonging to Saccharomyces, was used to trigger the fermentation because our previous works have shown its effectiveness in inducing beneficial changes in plant material [26,27,28,29]. 

The Neutral Red dye uptake (NR) and Alamar Blue (AB) assays showed that both non-fermented (EB) and fermented (FEB) extracts were not toxic to human skin cells; rather, they even stimulated metabolism and proliferation of fibroblasts and keratinocytes. The positive effect of EB and FEB on the proliferation of skin cells, both for HaCaT and BJ cells, is probably due to the high content of various phenolic compounds present in these samples (Table 1). These compounds have a proven positive effect on the proliferation and metabolic activity of skin cells, which is largely due to their strong antioxidant properties, resulting in a reduction in the level of oxidative stress in cells [30,31]. Phenolic compounds are found to inhibit the activity of proteinases, which catalyze the degradation of skin proteins, such as collagen and elastin [32]. Elderberry possesses ingredients favorable to cosmetic formulation, such as anthocyanins, which can reduce oxidative stress by scavenging free radicals, making them a potential anti-aging agent [33,34]. The gluconic acid present in the obtained ferments has a protective effect on skin cells by inhibiting the formation of ROS and DNA damage caused by single- and double-stranded breaks. It also reduces the activity of the inflammasome and IL-1β complexes [35]. The gallic acid present in the FEB also has protective properties against photoaging caused by UVB radiation, which has been proven using dermal fibroblasts in vitro and hairless mice in vivo. It is responsible for reducing the level of ROS, interlukin-6, and MMP-1, as well as for increasing the expression of type I procollagen [36]. As shown by Son et al. protocatechuic acid, present in both EB and FEB, exerts a strong antioxidant effect on human dermal fibroblasts treated with LPS, mainly through the ability to capture and scavenge ROS. In addition, this compound has the ability to reduce cell aging and to regulate the expression of type I collagen (COL1A1) and MMP1 genes [37]. The protective effect of this natural secondary metabolite is also related to the ability to absorb UVB, restore cellular redox balance, reduce oxidative damage to membrane lipids and DNA, and maintain the potential of the mitochondrial membrane. In addition, it has the ability to inhibit p65 NF-κB nuclear translocation and downregulate the expression of proteins associated with photoaging as well as of matrix metalloproteinases-1 and -9 and cyclooxygenase-2 [38]. *S. nigra* fruit extract can also protect skin cells from both the negative effects of UV radiation and bacterial LPS, thus limiting the inflammatory process [39]. The results obtained in this work showed the possibility of using *S. nigra* ferments obtained during fermentation with the use of kombucha as products characterized by broad biological activity and lack of cytotoxicity. The high potential of ferments obtained from various parts of plants has already been confirmed in many works of our group and other authors [26,28,40]. In addition, more and more research is focusing on fermented plant extracts, which are a rich source of antioxidants, vitamins, minerals, and polyphenols, as well as probiotics [41]. Due to the differences between the content of biologically active compounds between extracts and ferments from various plants, producing new ferments gives the opportunity to obtain cosmetic formulations that will have strictly defined properties adapted to the use of a specific product intended to eliminate various types of skin diseases [20].

Phytochemical investigation revealed that the fermentation process has significant impact on the qualitative and quantitative profile of polyphenols. Protocatechuic acid, followed by anthocyanins, was the most abundant in BE and FBE; however, the amount of protocatechuic acid (PA) increased, and that of cyanidin 3-sambubioside decreased, as a result of fermentation because PA is a major metabolite of cyanidin glycosides [42]. In addition, some phenolic compounds are formed, including chlorogenic acid, rutin, gallic acid, dihydroxybenzoic and hydroxybenzoic glucoside. Similar changes were observed during the fermentation of other plant material [26,27,28]. A significant amount of gluconic acid was also observed in the FEB, which is typically produced during carbohydrate metabolism [43].

Our study showed that both types of *S. nigra* extracts protect cells against LPS-induced cell damage in a similar manner. However, fermentation increased the anti-inflammatory potential, and FBE at concentration of 500 µg/mL was a more potent inhibitor of pro-inflammatory cytokines than EB at 1000 µg/mL. This could be attributed to the increased concentration of simple phenolics such as protocatechuic and chlorogenic acid. Protocatechuic acid (PA) is a desirable natural cosmetic ingredient because it exhibits anti-wrinkle and skin-whitening effects [44,45]. It also showed strong anti-inflammatory potential in different in vitro models and suppressed LPS-induced inflammation by affecting different metabolic pathways, including the SIRT1/ NF-kappaB and MAPK signaling pathways [46,47]. An in vivo study also confirmed its ability to alleviate inflammation, e.g., PA downregulated inflammatory markers such as IL-17, NF-κB, IKBKB, COX-2, and TNF-α in rats with induced pulmonary damage [48] and lowered the level of proinflammatory cyclooxygenase-2 in the brain and liver of rats with carbon tetrachloride-induced cytotoxicity [49]. PA is also a potent antioxidant, and it has been shown that it significantly decreases the level of malondialdehyde (MDA), a marker of lipid peroxidation, and increases the level of GSH and catalase, enzymes belonging to internal antioxidant system [48]. Chlorogenic acid also may participate in the anti-inflammatory activity of fermented extracts. Reports evidenced that it significantly attenuated LPS-induced COX-2, IL-6, IL-1ß and TNF-a expression [50] and enhanced the anti-inflammatory activity of curcumin in LPS-stimulated THP-1 cells [51]. There are also several papers describing the anti-inflammatory action of anthocyanins. For example, it has been found that cyanidin-3-glucoside reduced the levels of NO, PGE2, and IL-8 as well as the expression of iNOS and COX-2 in cytokine-induced inflammation in human intestinal cells [52]. Additionally, it inhibited the expression and secretion of TNF-α in stimulated human colon epithelial T84 cells [53].

In general, our study shows that the water extract and the bioferment from *S. nigra* fruits have beneficial properties that may positively affect the skin. They stimulate cell proliferation, inhibit collagenase and elastase, show ROS-scavenging activity and alleviate inflammation. Similar effects were also observed for other types of extracts from elderberries. Lin et al. found that an ethanolic extract attenuated UVB-induced ROS production, increased the resistance to oxidative damage, and downregulated the levels of IL-6 and VEGF in HaCaT cells. Furthermore, it blocked the overexpression of collagenase through inhibition of the MAPK/AP-1 signaling pathway and activated TGF-b/Smad, resulting in increased synthesis of collagen [39]. It also reduced NO production in LPS-stimulated macrophages and alleviated local inflammation caused by carrageenan paw oedema in rats [54]. An elderberry ethanolic extract and an extract obtained by supercritical fluid extraction demonstrated strong inhibitory effects on collagenase and elastase [54,55].

Our study has several limitations. First, the anti-inflammatory action was not assessed in HaCaT cells, where the effects could be different compared with fibroblasts. Secondly, a different model than LPS and TLR4-mediated signaling, such as H_2_O_2_-induced inflammation, could be more relevant in the context of skin aging. Therefore, further investigation of this activity is planned.

## 4. Materials and Methods

### 4.1. Plant Material and Sample Preparation

The plant material was purchased from Dary Natury company—a Polish producer and distributor of herbs (Grodzisk, Poland). Kombucha starter cultures were purchased from a commercial source in Poland. Extracts of *S. nigra* fruit were obtained by mixing 15 g of crushed fruit with 250 mL of distilled water at room temperature (approximately 20 °C). The extraction was carried out for 24 h on a magnetic stirrer. The extracts obtained were filtered twice using filters made of Whatman filter paper No. 10. The extract obtained was subjected to a fermentation process. For this purpose, 25 g of sucrose (final concentration 10.0% *m*/*v*) was added to the extract. Then 5 g of SCOBY and 25 mL of kombucha starter were added. Fermentation was carried out in sterile glass beakers (1000 mL, height 18 cm, diameter 8 cm) for a period of 14 days at room temperature (approximately 22 °C). At the end of the fermentation process, the resulting ferment was filtered through sterile gauze. The resulting extract and ferment were evaporated to dryness, diluted with purified water at room temperature (approximately 20 °C) to their final concentration and subjected to further analyses.

### 4.2. UHPLC-MS Analysis

All standards, including gallic acid, protocatechuic acid, chlorogenic acid, neochlorogenic acid, catechin, rutin, and cyanidin 3-glucoside and other reagents, namely MS-grade formic acid and MS-grade acetonitrile, were purchased from Sigma-Aldrich (St. Louis, MO, USA).

The extracts were separated using an ultra-high-performance liquid chromatograph (UHPLC) Infinity Series II with a DAD detector and an Agilent 6224 ESI/TOF mass detector (Agilent Technologies, Santa Clara, CA, USA) and an RP18 reversed-phase column Titan (Supelco, Sigma-Aldrich, Burlington, MA, USA) (10 cm × 2.1 mm i.d., 1.9 µm particle size). Water with 0.05% formic acid (solvent A) and acetonitrile with 0.05% formic acid (solvent B) were used as the mobile phases. The gradient program was as follows: 0–8 min from 98% A to 93% A (from 2% to 7% B), 8–15 min from 93% A to 88% A (from 7% to 12% B), 15–29 min from 88% A to 85% A (from 12% to 15% B), 29–40 min from 85% A to 80% A (from 15% B to 20% B), and 40–60 min from 80% A to 65% A (from 20% B to 35% B). The thermostat temperature was 30 °C, and The following ESI parameters were used: drying gas temperature 325 °C, drying gas flow 8 L min^-1^, nebulizer pressure 30 psi, capillary voltage 3500 V, skimmer voltage 65 V, and fragmentator voltage 180 V and 240 V. Ions were acquired from 100 to 1200 *m*/*z* in negative and positive modes. The MS identification was performed based on comparison with standards or with the literature when standards were not available. Quantification was based on calibration curves obtained using methanol standard solutions of the identified compounds. 

### 4.3. Antioxidative Activity

#### 4.3.1. DPPH Radical Scavenging Assay

In order to test the antioxidative activity of the tested ferments and extracts, the DPPH (1,1-diphenyl-2-picrylhydrazyl) radical was used. Samples at concentrations of 10, 50, 100, 250, 500, 1000, 1500 µg/mL were prepared and transferred to a 96-well plate (100 µL). Then 100 µL of a 4 mM methanolic DPPH solution was added and mixed thoroughly. Absorbance measurements at the wavelength λ = 517 nm were performed every 5 minutes for half an hour using a UV–VIS Filter Max 5 spectrophotometer (Thermo Fisher Scientific, Waltham, MA, USA). Water with a DPPH solution was used as a control. Measurements were repeated three times for each case. Based on the results obtained, the radical scavenging capacity of DPPH was calculated using the following Equation (1):

(1) $$\%\mathrm{DPPH}\;\mathrm{scavenging}=\frac{\mathrm{AC}-\mathrm{As}}{\mathrm{Ac}}\times 100$$

where As—absorbance of the sample; Ac—absorbance of the control sample. The obtained results were then used to calculate the IC_50_ value.

#### 4.3.2. ABTS+ Scavenging Assay

Another method for the determination of antioxidative properties was based on the use of the ABTS solution. First, a solution was prepared from 7 mM ABTS solution and 2.4 mM potassium persulfate solution mixed together in 1:1 ratio and then left at room temperature for not less than 14 hours. After this time, the resulting solution was diluted with methanol until the absorbance was about 1.0 (λ = 734 nm). Next, samples at concentrations of 10, 50, 100, 250, 500, 1000 µg/mL were prepared, and 1mL of each was mixed with 1 mL of the ABTS solution. Finally, the absorbance at λ = 734 nm was measured using the UV/VIS spectrophotometer Aquamate Helion (Thermo Fisher Scientific, Waltham, MA, USA). In this experiment, a solution of 1 mL of ABTS mixed with 1 mL of methanol was used as a control. Measurements were carried out in triplicate for each extract sample. The ABTS+ scavenging was calculated from Equation (2):

(2) $$\%\mathrm{of}\mathrm{ABTS}•+\mathrm{scavenging}=\frac{1-\mathrm{As}}{\mathrm{Ac}}\times 100$$

where As—absorbance of the sample; Ac—absorbance of the control sample. From the obtained results, the IC_50_ value was determined.

### 4.4. Cell Culture

Evaluation of the properties of the tested extract and ferment from *S. nigra* was performed on normal human keratinocyte (HaCaT, Cat. Number: 300493) and fibroblast (BJ, Cat. Number: CRL-2522) cells purchased from the American Type Culture Collection (Manassas, VA, USA). Both cell types were maintained in DMEM (Dulbecco Modified Basic Medium, Genos, Łódź, Poland) culture medium containing high glucose, supplemented with L-glutamine, sodium pyruvate, 5% FBS (Fetal Bovine Serum, Genos) and 1% antibiotics (100 U/mL penicillin and 1000 μg/mL streptomycin, Gibco, Waltham, MA USA). The cells were maintained at 37°C in a humidified atmosphere of 95% air and 5% carbon dioxide. After the test cells reached the appropriate confluence (approximately 70–80%), the culture medium was removed from the bottle and the cells were rinsed three times with sterile PBS (phosphate buffered saline, Genos). The cells were then trypsinized using 0.25%trypsin/EDTA (Gibco) solution and resuspended in fresh DMEM medium. After the cells had attached to the bottom of the culture bottles, they were exposed to the test samples. Fibroblasts were also additionally treated with LPS to assess the ability of EB and FEB to inhibit the inflammatory process.

#### 4.4.1. Cell Viability Test

##### Alamar Blue Assay (AB)

The first test used to evaluate the cytotoxicity of the *Sambucus nigra* extract and ferment toward skin cells was the Alamar Blue assay. This assay is based on the use of a resazurin-based solution (Sigma, R7017, Life Technologies, Bleiswijk, The Netherlands). The analyses were performed using the methodology previously described by Michalak et al. [56]. The assays were performed on two cell lines: keratinocytes (HaCaT) and fibroblasts (BJ), which were plated separately in transparent 96-well sterile flat-bottom plates (NEST Scientific, Woodbridge, NJ, USA) at a density of 1 × 10^4^ cells/well. The cytotoxicity of the analyzed samples (both extract and ferment dissolved in DMEM medium) was tested at concentrations of 100, 250, and 1000 μg/mL. The negative control was DMEM medium without the addition of elderberry extract or ferment. The test cells were exposed to the test samples for 24 h, after which the test solutions were aspirated and resazurin solution (60 mM) was added to each well. Plates prepared in this way were incubated in an incubator at 37 °C for 2 hours. After this time, the fluorescence of the samples placed in each well was measured. Measurements were made at an excitation wavelength of 530 nm and an emission wavelength of 590 nm using a microplate reader (FilterMax F5, Molecular Devices, Silicon Valley, CA, USA, Multi-Mode analysis software 3.4.0.25). HaCaT and BJ cells cultured separately in DMEM medium without the addition of extract or ferment were used as controls. For such cultured cells, 100% viability was assumed. As part of the work, three independent experiments were carried out, in which each extract and ferment concentration was tested in three replicates.

##### Neutral Red Assay (NR)

Cell cytotoxicity was also analyzed using a neutral red dye (Sigma Aldrich, St. Louis, MO, USA) according to the methodology previously described by Zagórska-Dziok et al. [57]. As in the case of the AB assay, both cell types were placed in 96-well plates and cultured for 24 hours. After the medium was aspirated, the cells were subjected to a 24-hour exposure to the tested samples at concentrations of 100, 250 and 1000 μg/mL. The negative control was DMEM medium without the addition of elderberry extract or ferment. After this time, the solutions of the tested samples were aspirated from the wells, and the cells were subjected to a 2-hour incubation with a solution of a neutral red dye (40 μg/mL). The cells were then washed with phosphate-buffered saline (PBS), and then a decolorizing solution (EtOH/AcCOOH/H_2_O_2_, 50%/1%/49%) was added. The prepared plates were shaken for 10 minutes, and then the uptake of the neutral red dye by the cells was measured by determining the optical density (OD) of the eluted dye at 540 nm in a FilterMax F5 microplate-reader spectrophotometer (Thermo Fisher). Three independent experiments were performed, during which each extract and ferment concentration was tested in triplicate. The results are presented as the percentage of the amount of dye retained compared with the control cells, for which a value of 100% was assumed.

### 4.5. Assessment of Matrix Metallopeptidase Inhibition

#### 4.5.1. Determination of Anti-Collagenase Activity

To assess the ability of the obtained extracts to inhibit collagenase activity, a fluorometric kit (Sigma-Aldrich, MAK293) was applied in accordance with the instructions attached to the kit and with the procedure described by Nizioł-Łukaszewska et al. [58]. A 10 mM solution of 1,10-phenanthroline (collagenase inhibitor) was used as a positive control. Collagenase Assay Buffer (CAB) was used as a negative control. The analyses were performed in a standard 96-well plate with a clear flat bottom. Absorbance was measured at a wavelength of 345 nm. The measurement was performed in the kinetic mode for 30 min at 37 °C. The ability of obtained extracts to inhibit COL activity was calculated using the following Equation (3):

(3) $$\%\mathrm{relative}\;\mathrm{COL}\;\mathrm{inhibition}=\frac{\mathrm{enzyme}\;\mathrm{control}-\mathrm{sample}}{\mathrm{enzyme}\;\mathrm{control}}\times 100$$

#### 4.5.2. Determination of Anti-Elastase Activity

To determine the possibility of inhibiting matrix metalloproteinases, a fluorometric neutrophil elastase (NE) kit (Sigma-Aldrich MAK246) was utilized in accordance with the attached instructions and the procedure described by Nizioł-Łukaszewska et al. [58]. Succinyl-alanyl-alanyl-prolyl-valine chloromethylketone (SPCK, elastase inhibitor) at a concentration of 20 mM was used as positive control. NE Assay Buffer with the test sample was used as a negative control. The analyses were performed in a standard 96-well plate with a clear flat bottom. Fluorescence was measured immediately at an excitation wavelength λ = 380 nm and emission λ = 500 nm using a microplate reader (FilterMax F5, Thermo Fisher Scientific, Waltham, MA, USA). The ability to inhibit the NE activity of the analyzed samples was calculated from the following Equation (4):

(4) $$\%\mathrm{relative}\;\mathrm{NE}\;\mathrm{activity}=\frac{∆\mathrm{RFU}\;\mathrm{test}\;\mathrm{inhibitor}}{∆\mathrm{RFU}\;\mathrm{enzyme}\;\mathrm{control}}\times 100$$

### 4.6. Anti-Inflammatory Assay

In order to assess the cytotoxicity and potential anti-inflammatory properties of the *S. nigra* extracts and ferments, fibroblasts were cultured under the conditions described above. Once they reached 70–80% confluence, they were pretreated (30 min) with samples of extracts (at concentrations of 500 and 1000 μg/mL) or standard compounds. After pretreatment, the cells were exposed to bacterial lipopolysaccharide (LPS) from *Escherichia coli* O111:B4 at a concentration of 10 μg/mL for a duration of 24 hours. Stock solutions of samples/compounds were prepared using DMSO/culture medium (1:1) and diluted to the working concentration. The final concentration of DMSO did not exceed 0.1%, and this concentration did not affect the cell viability. After 24-hour treatment of the cells with LPS, cytotoxicity and the level of the pro-inflammatory and anti-inflammatory interleukins IL-6, IL-8, IL-10, and TNF-α were measured immunoenzymatically by ELISA using commercially available kits (Elabscience, Houston, TX, USA) according to the manufacturer’s instruction. Three independent experiments were performed. 

### 4.7. Statistical Analysis 

Each value is the mean of three replicates. The values of the different parameters were expressed as the mean ± standard deviation (SD). The two-way analysis of variance (ANOVA) and Dunnett’s post hoc test between groups were performed. The statistical significance was determined at **** *p* < 0.0001, *** *p* < 0.001, ** *p* < 0.01, and * *p* < 0.05 compared with the control. Statistical analyses were performed using GraphPad Prism 8.4.3 (GraphPad Software, Inc., San Diego, CA, USA)

## 5. Conclusions

The results of the analyses indicate that the *S. nigra* fruit extract and its kombucha ferment are characterized by a significant content of biologically active compounds such as polyphenolic compounds belonging to phenolic acids, tannins and flavonoids, giving them antioxidant properties. The polyphenolic composition of the ferment was more diverse and richer in some compounds compared to the extract. In vitro studies using skin cells—fibroblasts and keratinocytes—showed that both the extracts and the ferments had a positive effect on skin cell viability and metabolism. However, a more beneficial effect was observed with the ferment. The kombucha extract and ferment showed an inhibitory effect on elastase and collagenase enzymes. This may indicate that they can be used in cosmetics as substances to slow down the aging process. Furthermore, the study showed that both the extract and the ferment modulate levels of inflammatory factors. Due to their multidirectional activity, they may help in the prevention of many skin conditions. In addition, the literature data indicate that the development of fermented plant extracts has great potential in the cosmetic industry, because, in addition to their probiotic activity supporting beneficial microorganisms inhabiting human skin, they can also be a valuable ingredient in pharmaceutical and cosmetic products.

## Figures and Tables

**Table 1:** Mass data and results of quantitative analysis (±SD) of polyphenols in the extract and the fermented extract from fruit of *S. nigra*.

Rt (min)	Observed Ion Mass [M-H]-/(Fragments)	Δ ppm	Formula	Identified	Extract (µg/g)	Ferment (µg/g)
1.49	195.05182	4.05	C_6_H_12_O_7_	Gluconic acid *	-	+++
3.44	169.01431 (125)	0.37	C_7_H_6_O_5_	Gallic acid *	-	4.3 ± 0.3
4.32	299.0778 (137)	1.86	C_13_H_16_O_8_	Hydroxybenzoic acid hexoside	-	+
6.32	153.01946	0.83	C_7_H_6_O_4_	Protocatechuic acid *	33.2 ± 1.5	181.1 ± 9.8
7.11	315.07(153)	2.4	C_13_H_16_O_9_	Dihydroxybenzoic hexoside	-	5.8 ± 0.2
8.69	353.08798 (191,179)	0.49	C_16_H_18_O_9_	Neochlorogenic *	-	12.8 ± 0.7
12.77	741.18864 (285)	0.37	C_32_H_38_O_20_	Cyanidin 3-sambubioside-5-glucoside	+	-
14.29	353.08804 (191,179)	0.66	C_16_H_18_O_9_	Chlorogenic *	0.4 ± 0.1	41.3 ± 2.1
13.05	289.07189	0.44	C_15_H_14_O_6_	Catechin *	-	+
13.21	579.13567 (285)	0.22	C_26_H_28_O_15_	Cyanidin 3-sambubioside	42.3 ± 3.4	27.4 ± 1.8
18.29	447.09354 (285)	0.57	C_21_H_20_O_11_	Cyanidin 3-glucoside *	34.3 ± 1.9	37.4 ± 2.1
21.09	525.19764 (327)	−0.21	C_25_H_34_O_12_	unidentified	+	+
29.26	609.14650	0.64	C_27_H_30_O_16_	Rutoside *	-	+
36.22	610.42488	1.62	C_38_H_59_O_6_	unidentified	+++	+
43.59	723.50515	−0.17	C_41_H_72_O_10_	unidentified	+++	+
48.66	836.58698	0.39	C_44_H_85_O_14_	unidentified	+++	+

* identification was confirmed using standards; - not detected; + detected; +++ present at high concentration.

**Table 2:** Values of IC_50_ of DPPH and ABTS radical scavenging for *Sambucus nigra* L. extract and kombucha ferment. Values are means ± standard deviation (SD) of triplicates.

Method	Extract	Ferment
	IC_50_ [ µg/mL]	IC_50_ [µg/mL]
DPPH	865 ± 1.2 ****	909 ± 1.4 ****
ABTS	91 ± 0.45 ****	95 ± 0.65 ****

**** denotes significant differences between the values, with *p* < 0.0001.

## Data Availability

The data presented in this study are available on request from the corresponding author.

## Supplementary Materials

The following supporting information can be downloaded at: https://www.mdpi.com/article/10.3390/ijms241210286/s1.
