# Supplementary material for: Anti-Inflammatory and Protective Effects of Water Extract and Bioferment from Sambucus nigra Fruit in LPS-Induced Human Skin Fibroblasts
Source: Int J Mol Sci. 2023 Jun 17;24(12):10286. doi: 10.3390/ijms241210286 (PMC10299406; doi:10.3390/ijms241210286)
Supplement: Supplementary file 1 [file ijms-24-10286-s001.zip › ijms-2454239-supplementary.pdf]

International Journal of *Molecular Sciences*

MDPI

Supplementary Materials

Anti-Inflammatory and Protective Effects ofWater Extract and Bioferment from Sambucus nigra Fruit in LPS-Induced Human Skin Fibroblasts

Magdalena Wójciak [1], Aleksandra Ziemlewska[2], Martyna Zagórska-Dziok[2], Zofia Nizioł-Łukaszewska[2], Dariusz Szczepanek [3], Tomasz Oniszczuk [4]* and Ireneusz Sowa[1]*

1 Department of Analytical Chemistry, Medical University of Lublin, Chodzki 4a, 20-093 Lublin, Poland; magdalena.wojciak@umlub.pl
2 Department of Technology of Cosmetic and Pharmaceutical Products, Medical College, University of Information Technology andManagement in Rzeszow, Kielnarowa 386a, 36-020 Tyczyn, Poland; aziemlewska@wsiz.edu.pl (A.Z.); mzagorska@wsiz.edu.pl (M.Z.-D.); zniziol@wsiz.edu.pl (Z.N.-Ł.)
3 Chair and Department of Neurosurgery and Paediatric Neurosurgery, Medical University of Lublin, 20-090 Lublin, Poland; dariusz.szczepanek@umlub.pl
4 Department of Thermal Technology and Food Process Engineering, University of Life Sciences in Lublin, Głeboka 31, 20-612 Lublin, Poland
* Correspondence: tomasz.oniszczuk@up.lublin.pl (T.O.); i.sowa@umlub.pl (I.S.)

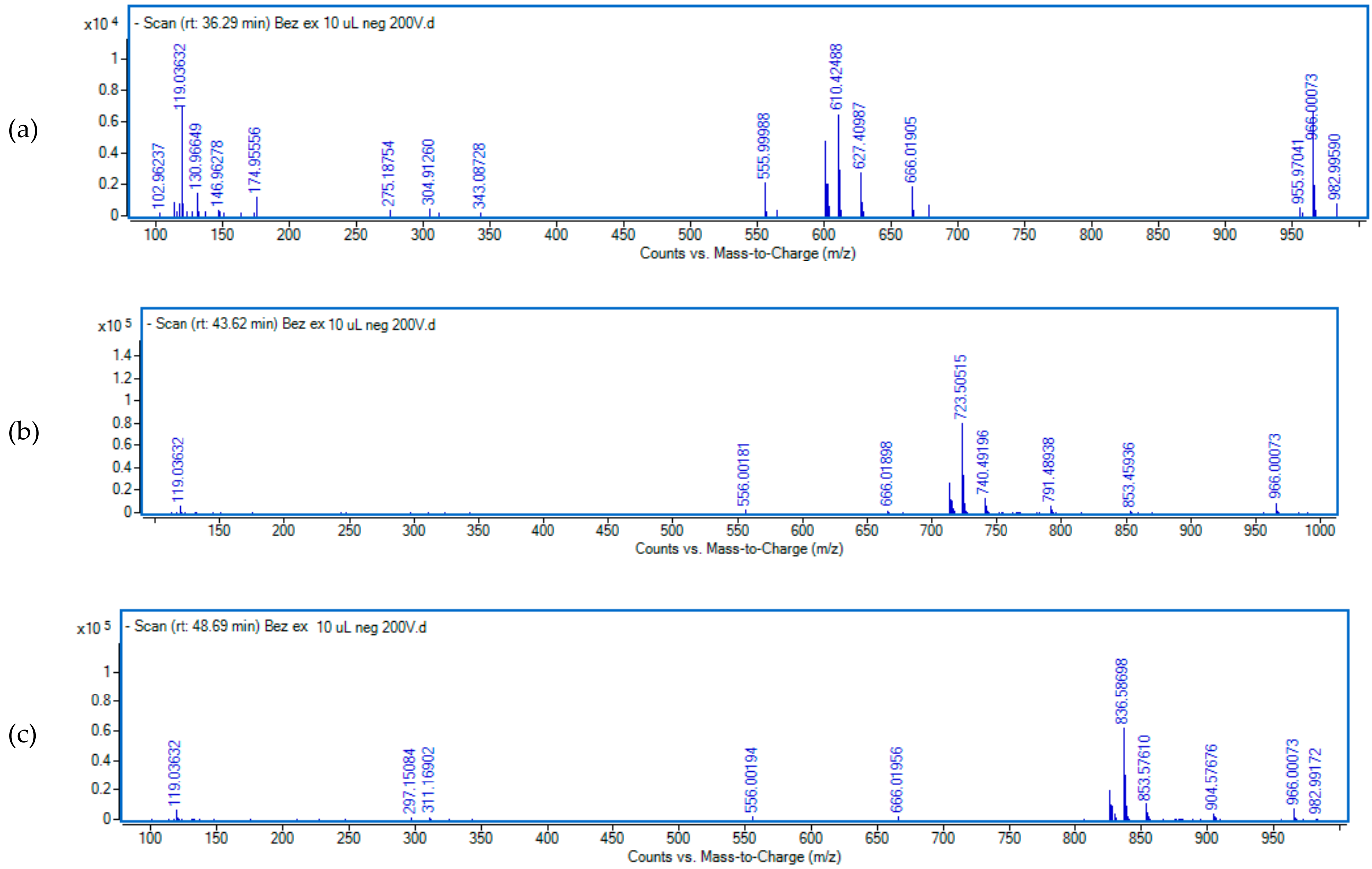

Figure S1. Representative MS spectra of unknown components found in *S. nigra* fruit extract. MS data are given in Table 1. (**a**) peak at retention time 36.29 (**b**) peak at retention time 43.62 (**c**) peak at retention time 48.69.

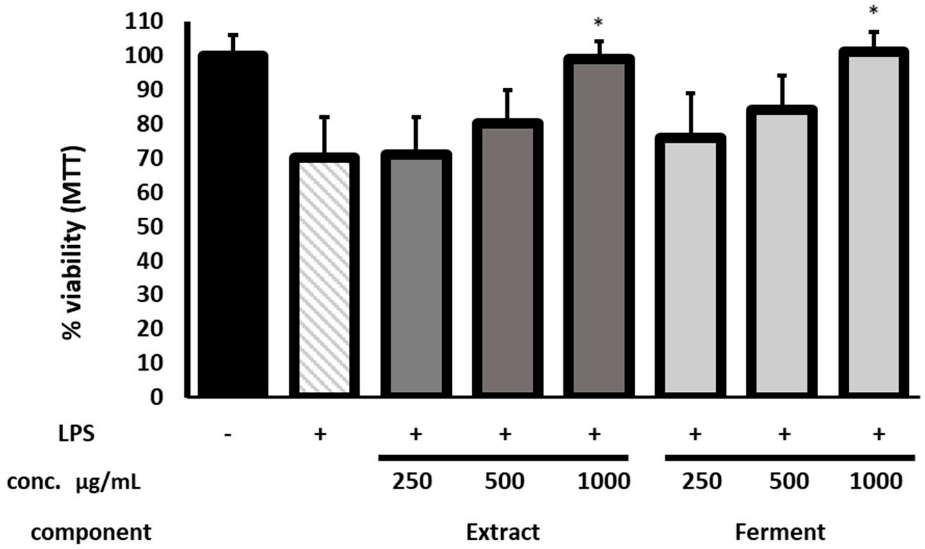

Figure S2. Effect of pretreatment with different concentrations of *S. nigra* fruit extract (EB), fermented extract (FEB) prior to LPS exposure on cells viability - MTT assay. The data represent means ± SD (n = 3). * indicates a statistically significant difference ($p < 0.05$) compared to the LPS-treated cells. One-way ANOVA followed by Dunnett's multiple comparison post hoc test.
